# Neurorétinite stellaire révélant une bartonellose

**DOI:** 10.11604/pamj.2014.19.344.4017

**Published:** 2014-12-03

**Authors:** Rajae Derrar, Rajae Daoudi

**Affiliations:** 1 Université Mohammed V Souissi, Service d'Ophtalmologie A Hôpital des Spécialités CHU Rabat, Maroc

**Keywords:** Neurorétinite stellaire, bartonellose, acuité visuelle, Stellar neuroretinitis, bartonellosis, visual acuity

## Image en medicine

Patient âgé de 22 ans admis pour baisse d'acuité visuelle d'installation rapidement progressive de l’œil gauche sans notion de traumatisme. L'interrogatoire révèle la notion de griffure par chat domestique du pied gauche 3 mois auparavant avec syndrome grippal dans la semaine qui a suivi et adénopathies inguinales inflammatoires ayant régressé spontanément au bout de 2 mois. A l'admission, l'acuité visuelle au niveau de l’œil gauche était de 3/10, et 10/10 au niveau de l’œil droit. L'examen note un segment antérieur calme avec au fond d’œil un œdème papillaire (flèche blanche) associé à des exsudats maculaires disposés en étoile (flèches noires). Le reste de l'examen ophtalmologique est normal. L'examen général note une cicatrice au niveau du pied gauche. Devant cet aspect, une bartonellose a été fortement suspectée, un bilan comportant un examen interniste, une sérologie de lyme, une IDR à la tuberculine et une sérologie de bartonella a été demandé. Cette dernière a révélé un taux élevé d'Ig G antiBartonella Hensalae. Devant la certitude diagnostique de la bartonellose, un traitement à base de doxcicycline pendant 1 mois à raison de 200mg/j associé à une corticothérapie par voie générale à une dose de 1mg/kg/Jour pendant 5 jours a été instauré amenant à une régression de l'oedeme papillaire ainsi qu'a la récupération d'une bonne acuité visuelle. La régression des exsudats était plus lente. La bartonellose est une pathologie infectieuse secondaire à une bactérie intracellulaire facultative nommée bartonella hensalae est essentiellement transmise par une morsure ou griffure de chats.

**Figure 1:**
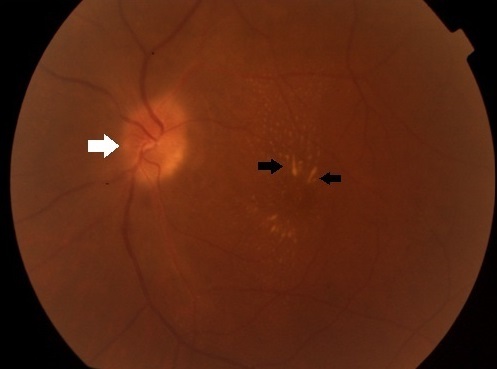
Aspect du fond d’œil montrant un oedeme papillaire (flèche blanche) avec des exsudats maculaires organisés en étoile (flèches noires)

